# A New Method of Starting Gold Fillings

**Published:** 1906-02

**Authors:** W. L. Schreiber

**Affiliations:** Philadelphia, Pa.


### A NEW METHOD OF STARTING GOLD FILLINGS.

By Dr. W. L. Schreiber, Philadelphia, Pa.

The latest method which I have been using in the preparation of cavities to be filled with gold, suggested itself to me through the use of the Jenkins' inlay system.

It consists in making the cavity retentive, but without the deep grooving or retaining-pit, making just enough undercut to retain the finished filling. I then take a piece of No. 30 gold-foil, which is used in the Jenkins' system, large enough to suit the case. Then proceed the same as in making the matrix for a porcelain inlay, pressing the foil in absolute contact with the cavity walls and margins, the latter being beveled as is usually done for a gold filling, the excess being pressed out over the exposed surfaces of the tooth, *i. e.,* the enamel. It may sometimes be necessary to ligate the matrix in until you have partially filled the cavity, but I have never found this necessary, because of the close adaptation of the foil to all surfaces. Those of you who have experienced the difficulty which attends the removal of a properly-prepared matrix, even where no undercuts exist, will readily see how this matrix will keep its place throughout the operation. I then pack soft gold into the slight undercuts and upon the floor and walls of the cavity, finishing with cohesive.

This method does away with the use of retaining pits and the use of varnishes to start the filling, as advocated by many practitioners and sold by dental supply houses, although I have never found the necessity to use them. Only the smallest amount of undercutting is necessary if made in the right place, thereby reducing the encroachment upon the pulp to a minimum, which must be acknowledged to be a great consideration, especially in deep-seated cavities.

The exposed surface of the matrix should be wiped off with alcohol or chloroform and dried with hot air before commencing the packing of the gold.—*Summary.*

